# Highly Nutritional Bread with Partial Replacement of Wheat by Amaranth and Orange Sweet Potato

**DOI:** 10.3390/foods11101473

**Published:** 2022-05-19

**Authors:** Ana M. Calderón de la Barca, Luz E. Mercado-Gómez, Nina G. Heredia-Sandoval, Valeria Luna-Alcocer, Patricia M. A. Porras Loaiza, Humberto González-Ríos, Alma R. Islas-Rubio

**Affiliations:** 1 Centro de Investigación en Alimentación y Desarrollo, A.C., Hermosillo 83304, Sonora, Mexico; luz.mercado.mc19@estudiantes.ciad.mx (L.E.M.-G.); nina_heredia@hotmail.com (N.G.H.-S.); alcocer_vale@hotmail.com (V.L.-A.); hugory@ciad.mx (H.G.-R.); aislas@ciad.mx (A.R.I.-R.); 2 Esc. Negocios y Economía, Universidad de las Américas Puebla, Cholula 72810, Puebla, Mexico; patricia.porras@udlap.mx

**Keywords:** bread, wheat, amaranth, orange sweet potato, optimized blend

## Abstract

The current dietary habits cause health problems due to foods’ composition, with bread as an important example. Our aim was to formulate an optimum dough blend with flours from wheat, amaranth and orange sweet potato to obtain a physically good and highly nutritional bread. Bread was prepared with blends of wheat, amaranth and orange sweet potato flours, optimizing the technological properties of the doughs by the response surface methodology and analyzing their physical and nutritional properties. Amaranth provides protein and fiber, and sweet potatoes provide β-carotenoids and high antioxidant activity. The prediction models were adjusted by mixing time (MT), peak dough resistance (PDR), setback (SB) and breakdown (BD). The interaction between wheat and amaranth significantly (*p* < 0.05) affected MT, PDR and SB, while the interaction between amaranth and sweet potato affected BD (*p* < 0.05); none of the components influenced PDR. The optimized blend (68.7% wheat, 22.7% amaranth and 8.6% sweet potato) produced a bread with the best crust and crumb appearance. This bread was comparable to that made with 100% wheat in specific volume and textural characteristics, but had better protein quality, higher content of fermentable fiber, pro-vitamin A, and bioactive compounds with good antioxidant capacity, and a lower glycemic index.

## 1. Introduction

The rich Mesoamerican ancestral diet included ingredients such as amaranth and sweet potato in bakery products of high nutritional and functional quality [1]. The amaranth grain is an excellent possibility for new recipes because of its high-quality proteins and high content of minerals and dietary fibers [2,3]. Although sweet potato has been used since old times, the orange sweet potato is a novel variety with an even richer content of bioactive compounds such as phenolics and β-carotenes, with antioxidant properties [4].

The dietary habits have changed worldwide, promoting overweight and obesity due to the high caloric intake associated with a low physical activity level. Among the foods with a high contribution to energy are bread and pastries or sweet bakery products [5] with a high content of fat and sugar and a low fiber content [6]; these are additionally, the more common takeaway products, from Latin America to East Europe [5,6]. Therefore, a change in the composition of bread and pastries could help to prevent excessive weight gain and diet-related diseases and promote a healthy nutrition. The modification should be subtle to maintain physical characteristics similar to those of the highly accepted wheat-made bread while providing increased nutritional quality.

It is well known that a total or partial substitution of wheat flour with its ideal properties for bread-making could induce negative changes in dough properties and crumb and crust appearance. Even a 15% amaranth flour replacement of wheat flour negatively affected the physical and textural characteristics of bread, despite improving its nutritional quality [7]. We prepared a 100% amaranth bread with good physical properties but with quite different appearance and sensorial properties compared to wheat bread [3].

Orange sweet potatoes have been used also to prepare bread. A simple partial replacement of wheat flour by sweet potato puree produced bread with good nutritional qualities [8]. In Nigeria, a very complex composite flour with sweet potatoes, soy concentrate, date palm powder and potato starch was prepared [4]; the bread presented a high content of β-carotene and a low glycemic index. No information about its physical and textural properties was provided in the two former studies. Two Ghanaian-style breads, with partial substitution of wheat flour with a sweet potato puree, including butter and milk, were prepared and resulted to have high acceptability and a high β-carotene content [9], but they could not replace the occidental wheat bread.

Our objective was to formulate an optimum blend of flours from wheat, amaranth and orange sweet potato to obtain dough with the best properties to prepare bread with similar physical properties to those of wheat bread. Additionally, we aimed to obtain better nutritional qualities due to macronutrients, β-carotenes and total phenols as well as optimal antioxidant capacity, amino acid score, fermentable fiber functionality and glycemic index in vitro.

## 2. Materials and Methods

### 2.1. Materials

Popped amaranth seeds (*Amaranthus hypochondriacus* L.) were from an indigenous community in San Juan de Amecac, Puebla, Mexico. Orange sweet potatoes (*Ipomoea batatas*), wheat flour, yeast, salt and sugar were from a local market. Chemical-grade reagents were from Sigma Aldrich.

### 2.2. Flour Preparation and Property Analysis

The popped amaranth seeds were ground in a Pulvex 200 grinder (Molinos Pulvex, Mexico City, Mexico) and sieved through a 250 μm mesh (US No. 60) in a Dura Tap sieve shaker (DT168 Advantech Mfg. Co., New Berlin, WI, USA). The orange sweet potatoes were peeled, cut into 2 cm cubes, blanched in boiling water for 5 s and dried in a Riossa E-33 oven (Mexico City, Mexico) at 50 °C for 10 h. After drying, they were ground and sieved as described for amaranth.

#### 2.2.1. Proximate Analysis and Bioactive Compounds

Moisture (method 44-15A) and protein content (method 46-30B) were determined according to the AACC [10]; total lipids (method 920.39) and ash (method 923.03) were determined by AOAC methods [11]. Carbohydrates were calculated by difference. Total phenolic compounds were analyzed according to [12]. DPPH scavenging capacity was measured to determine the antioxidant activity [13]. Carotenoids were quantified by HPLC [14]. All analyses were performed in triplicate.

#### 2.2.2. Viscosity and Mixographic Measurements

RVA analysis was performed following the AACC method 76-21 [10]. Each flour blend (3.5 ± 0.1 g, 14% moisture basis) was mixed with 25 ± 0.1 g of water, heated to 50 °C, brought to 95 °C, kept at this temperature for 2.5 min, then cooled to 50 °C for 2 min; setback (SB) and breakdown (BD) were obtained. Analyses were carried out in triplicate.

The mixographic measurements were performed on 30 g (14% moisture basis) of each blend using a mixograph (model 9T51B508, National Manufacturing Co., Lincoln, NE, USA), at 25 °C according to the AACC method 44-40A [10]. Mixing time (MT) and peak dough resistance (PDR) were graphically evaluated.

### 2.3. Flour Blend Optimization and Breadmaking

The amount of each flour (wheat, amaranth and sweet potato) was estimated using a central composite rotational design with 3 factors, performing 18 experimental runs. The proportion of wheat flour (X_1_) ranged from 50% to 70%, that of amaranth flour (X_2_) from 10% to 30%, and that of sweet potato flour (X_3_) from 5% to 10%, as shown in Table 1. Breads were elaborated with the optimum blends as well as with 100% wheat flour as a control (two replicates for each bread). For the control bread, we used the 10-10B method [10], with 100 g of wheat flour, 1.5 g of salt, 2 g of yeast, 6 g of sugar, 3.43 g of fat and 54.28 mL of water, considering the protein content of each blend at a 14% moisture basis and the mixographic behavior.

For the breads prepared with the optimum blends instead of wheat alone, we omitted punches, and they were molded just after mixing. For the final bread, sugar was reduced by 50% (3 g). All doughs were fermented for 90 min.

### 2.4. Physical and Nutritional Composition of the Breads

Bread volume (cm^3^) was evaluated by the rapeseed displacement method, and the specific volume was calculated in relation to weight (cm^3^/g). The texture analysis used slides at 1.7 mm/s to compress the breads’ crumb center to 50% of their original height. The parameters hardness, cohesiveness and chewiness [15] were measured by a double compression test using a texturometer (TA-XT2, Stable Micro Systems Ltd., Godalming, Surrey, UK). Three replicates of each measurement were examined.

The amino acid profile was analyzed by HPLC [16] in triplicate samples after acid digestion and derivatization with ο−ftalaldehide, in a Thermo Scientific Ultimate 3000 chromatograph with a C18 column Microsorb 100-3, and detection was performed by fluorescence. The glycemic index was determined in vitro using a modification of the method of Goñi et al. [17], with respect to glucose. Glucose was quantified by the D-Glucose assay kit (GOPOD format) from Megazyme (K-GLUC). Dietary fiber was quantified using a Megazyme kit (K-TDFR-200A).

Fiber in the optimal flour blend was fermented in vitro after gastrointestinal digestion, in comparison to that of wheat flour, according to [18] with some modifications. Digestion in vitro was carried out at 37 °C with pepsin and pancreatin for 1 and 6 h, respectively, and pancreatic amylase for 16 h. After centrifugation, the supernatant was evaluated for the degree of hydrolysis and glucose content. The precipitates (fibrous residues) were analyzed for moisture and nitrogen [10], total phenols and flavonoids [12]. Each fibrous residue was used as a substrate for in vitro fermentation by the action of the microbiota from the homogenized human feces of 10 healthy volunteers with healthy or ultra-processed dietary patterns. The fecal homogenates were added to sterile medium plus each fibrous residue; incubation was carried out in a 5% CO_2_ chamber at 37 °C [18]. Aliquots of each fermentation were taken at 0, 12 and 24 h. *Bacteroides* spp. and *Prevotella* spp., the two bacterial genera more common in the microbiota of the study population, were quantified as a proportion of total bacterial DNA. Amplification of the bacterial 16S rRNA was performed with the standard curve protocol in a StepOnePlus PCR system [19].

### 2.5. Statistical Analysis

We analyzed the values of the variables MT, PDR, SB and BD by response surface methodology, fitting a quadratic model with the JMP 13.10 software (SAS Institute Inc., Cary, NC, USA). The second-order polynomial equation included the linear, quadratic and interaction effects of the factors wheat, amaranth and sweet potato. Significant terms of the model were found by analysis of variance (ANOVA) for each response. Lack of the fit and determination coefficient (adjusted R^2^) were calculated to verify the accuracy and adequacy of the model. Quadratic terms of the factors were not significant for the full fitted model; therefore, they were moved out of the model. Additionally, 3-D surface plots were generated with the same software. After obtaining the predictive models, the optimal values of the independent variables for the optimum response were estimated using the desirability function available from the response surface methodology. The criterion was a maximum response for PDR and minimal ones for MT, SB and BD. One-way ANOVA was applied for the proximate composition of flours and other variables of the obtained breads and the Tukey–Kramer test for means comparison (or the Kruskal–Wallis test for non-normal variables). Significance was considered at *p* < 0.05.

## 3. Results and Discussion

Table 2 presents the proximate composition of the wheat, amaranth and sweet potato flours. Amaranth contained a higher level (*p* < 0.05) of protein than wheat, in addition to minerals (as ashes in Table 2) and 15.73 ± 0.13% of dietary fiber as part of its carbohydrates. Although the orange sweet potato appears poor in macronutrients, its starches reduce the glycemic index of composite breads [20]. Additionally, this potato contains 14,692 ± 241 µg/100 g of β-carotenes and 235 ± 0.2 mg GAE/100 g of total polyphenols and has an antioxidant capacity of 21.2 ± 5.3 µmol TE/g. The orange sweet potato is an excellent source of phenolic compounds, with high antioxidant activity [4]. Although wheat bran contains polyphenols with antioxidant capacity, the refined flour is not a good source of them and does not contain β-carotenes.

### 3.1. Optimized Blends for Breadmaking

The prediction models were adjusted by MT, PDR, SB and BD. Quadratic factors were lost by adjusting; therefore, only linear and combined interactions remained. The interaction between wheat (X_1_) and amaranth (X_2_) significantly affected (*p* < 0.05) MT, PDR and SB, explaining 73%, 79% and 76% of the variation, respectively. In addition, the linear terms (X_1_, X_2_ and X_3_) were significant for PDR (*p* < 0.05). The interaction of wheat (X_1_) and amaranth (X_2_) was negative for MT and PDR, while the same interaction was positive for SB (*p* < 0.05). Finally, the interaction of amaranth (X_2_) and sweet potato (X_3_) was significantly positive for BD (*p* < 0.05), with the highest R^2^ value and 86% of the variability explained by the proposed model (Table 3).

The PDR behavior is important due to its direct correlation with the specific volume of the produced bread [21]; conversely, MT is indirectly related to dough extensibility [22]. SB and BD are related to starch retrogradation and bread hardness, with low values being positive for bread quality with respect to slow hardening and better stability [23]. Therefore, these parameters are clear indicators of the bread-making quality.

**Figure 1:**
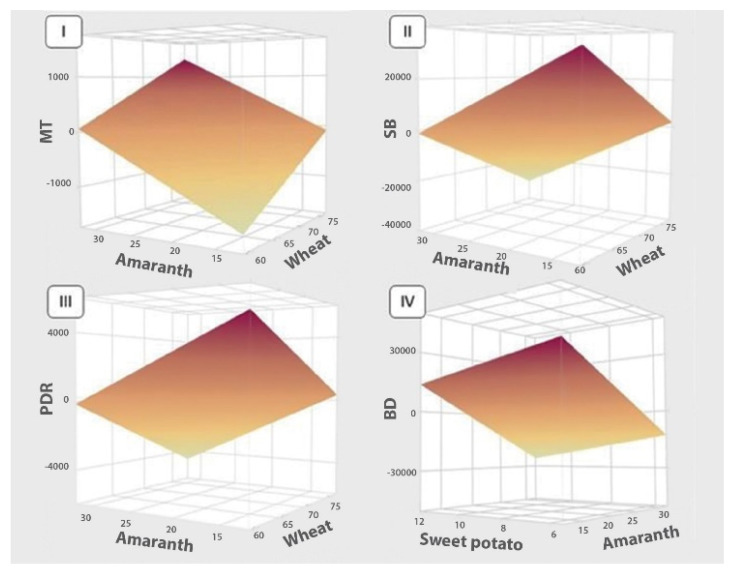
Determinant ingredients for the response variables: MT, mixing time; PDR, peak dough resistance; SB, set back; BD, breakdown.

Figure 1 shows the determinant ingredients for each variable’s response in addition to their relationship to each other, all of them with a linear behavior. In panels I, II and III corresponding to MT, SB and PDR, respectively, it is possible to see the interaction of wheat with amaranth, which was significant for the three variables. Panel II shows that blends containing less than 65% of wheat and 15% of amaranth were outside of the predictor. Figure 1 (IV) presents the variable BD, which was only significant for the interaction between sweet potato and amaranth, where amaranth had a higher influence on the response changes. It is interesting to see that SB, PDR and BD in Figure 1 (II, III and IV) were negative when more than 30% of amaranth was added, although only for PDR the change was significant.

After optimization, four blends were obtained with 63–76% wheat, 13–30% amaranth and 7–11% sweet potato flours (Table 4). Blends 1 and 2 were designed from the estimator model of each variable; blend 3 was obtained from the central points of the range for each ingredient. When searching for a blend with less than 68% wheat and a PDR higher than 33, blend 4 appeared to satisfy these criteria, as shown in the design matrix. The PDR values of the four blends ranged from 33 to 40, among the higher values of the matrix. The highest MT value was 5 min, SB was low in all cases, and BD values ranged from 56 to 248, with 56 determined for blend 4.

### 3.2. Breadmaking and Product Characterization

**Figure 2:**
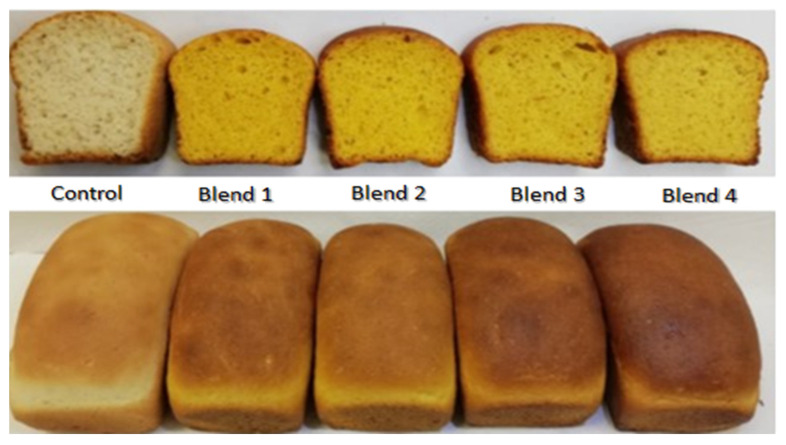
Appearance of the crusts and crumbs of the loaves made with the optimum blends of wheat, amaranth and orange sweet potato flours.

Bread was prepared using the four optimized blends, in addition to 100% wheat as a control. The resulting loaves of the optimum blends of wheat, amaranth and orange sweet potato were good. Figure 2 shows the obtained crusts, smooth and without cracks. The crumb appearance was uniform, with a yellow color because of the orange sweet potato content. Clearly, it was an advantage to optimize the blends by the response surface methodology, because no chemical additives were needed to obtain a good appearance in spite of the even more than 30% substitution of wheat flour that caused a partial loss of the functional gluten. Studies about bread elaboration with composite flours used a high degree of orange sweet potato flour substitution [4,9]. They did not present information about the resulting bread’s physical characteristics but a general description, because their attempts were to contribute to reducing nutritional deficiencies in protein and vitamin A. In the present study, the attempt was to offer a bread for sandwiches similar in appearance but with better nutritional quality than the 100% wheat-containing commercial breads.

The different bread loaves prepared with the optimum blends were comparable in textural characteristics to that made with 100% wheat flour, as shown in Table 5. However, the breads made with blends 1 and 2 presented higher values for hardness and chewiness than those of blends 3 and 4, and blend 3 was preferable to blend 4 because of a better cohesiveness. Therefore, the bread with blend 3 was chosen for composition analyses. These results are important to ensure the acceptability of the new highly nutritive bread, especially by schoolchildren who may consume it in their sandwiches.

### 3.3. Nutritional Composition of the Produced Bread

The proximate composition of the bread prepared with blend 3 is shown in Table 6, with similarities and differences with respect to the bread prepared with 100% wheat flour. There was no change (*p* > 0.05) in protein content, but the quality was different as explained in the next paragraph. The bread from blend 3 contained a higher (*p* < 0.05) amount of ash and lipids than the 100% wheat bread, because of the amaranth grain presence. Although the total carbohydrates appeared to be exactly the same in proportion, we found a difference in dietary fiber, as described below.

For healthy nutrition, the quantity of protein is very important as well as its quality, evaluated on the basis of the indispensable amino acid profile and digestibility. A simple evaluation of protein nutritional quality consists in quantifying the indispensable amino acids and in calculating their proportion with respect to the recommended profile [24]. The smallest rate value corresponds to the limiting amino acid and is called amino acid score. Table 7 presents the indispensable amino acid profile of the protein in the bread from blend 3; it exceeded the recommendation for almost all the indispensable amino acids, except for Leu and Lys, although the limiting amino acid appeared to be Lys, with a score of 62 (or 0.62). Wheat protein showed a lower quality, with 0.49 for Lys, as the limiting amino acid. Additionally, the digestibility of wheat protein was lower than that of the blend 3 protein, as its degree of protein hydrolysis was 19% lower than that of blend 3, as evaluated after the in vitro pepsin/pancreatin assay (assay for fiber fermentation). Due to the high content of proline in gluten proteins, gastrointestinal enzymes are not able to cleave part of these sequences [25].

Because of its composition, the bread prepared with blend 3 provides dietary fiber, β-carotenes and other compounds with antioxidant capacity. In addition, its glycemic index is lower than that of conventional wheat bread. The results are shown in Table 8.

A very important quality of the bread made with blend 3 is its high dietary fiber content, i.e., 4.98 g/100 g of bread in comparison to 2.04 g/100 g in wheat bread; this characteristic is attributed to the high fiber content in amaranth. Currently, it is possible to find several expensive commercial breads with wheat bran on their crust to suggest a high fiber content. Such strategy is not a healthy advantage because it cannot mitigate the adverse consequences of obesity as fermentable fibers do [27]. The fiber of blend 3 was more fermentable, as described below, than that of wheat. The high fiber content is not detectable by the tongue, which could increase the bread acceptability by children.

After 12 h of fermentation in vitro, *Bacteroides* and *Prevotella* remained in almost the same proportions with respect to total bacteria when the source of fiber was 100% wheat flour. In contrast, *Bacteroides* increased significantly (*p* < 0.05) after 12 h of fermentation when the source of fiber was the blend 3 flour, while *Prevotella* levels remained similar (*p* > 0.05). The important properties of fiber to sustain functions in the organism include its capacity to react with water, its colligative property, its capacity to ferment and to chelate [28]. Such functions influence the metabolism directly or through the microbiota. Although the fiber content of blend 3 was 5:1 insoluble:soluble, *Prevotella* did not increase but *Bacteroides* did. It is possible that the Bacteroidetes composition was affected by the content of total phenols in the fibrous residues of the bread made with blend 3.

With respect to total phenols and β-carotenes (Table 8), the baking process induced losses. Oloniyo et al. [4] prepared blends with flours of orange sweet potato, soy concentrate, date palm and potato starch, and processing significantly affected the β-carotene content. Our results shown in Table 8 for β-carotenes and total phenols correspond to these previous results [4] if the proportions are considered.

In spite of the antioxidant activity evaluated as TEAC or DPPH being affected by processing, especially the long fermentation time and the high baking temperature, when it was measured as ORAC, the value increased with respect to the original one. Possibly, the phenolic compounds can be better detected by their ability to neutralize free peroxyl radicals as ORAC does [29]. The glycemic index is a very important parameter for healthy nutrition. It evaluates food carbohydrates from 0 to 100 as a measure of the velocity of glucose increasing in the circulation of the consumers after eating [30]. The glycemic index of the blend 3 bread was 51.8, a value that is considered low (≤55), and 27% lower than that of the 100% wheat bread with a high index (≥70). In spite of differences in composition, our blend 3 bread compares very well in glycemic index with those prepared by Oloniyo et al. [4] with a different blend. Multiplying the value of the glycemic index as a percentage by the available carbohydrates (without fiber) results in the glycemic load. Therefore, a slice (26 g) of blend 3 bread has a glycemic load of 5.7, while a comparable slice of 100% wheat bread has a glycemic load of 8.1. At the end, the blend 3 bread appeared to contain less digestible carbohydrates than the wheat bread, which contribute to energy consumption and eventually to excessive body weight gain.

## 4. Conclusions

The optimized blend 3 with 68.7% wheat, 22.7% amaranth and 8.6% sweet potato, produced a bread with the best crust and crumb appearance. This bread was comparable to that made with 100% wheat in specific volume and textural characteristics, but had a better protein quality, higher content of fermentable fiber, pro-vitamin A, and bioactive compounds with good antioxidant capacity, and a lower glycemic index.

## Figures and Tables

**Table 1:** Matrix of the central composite rotational design and experimental values of the parameters of the blends with percentages of wheat, popped amaranth and orange sweet potato.

Blend	Wheat	Amaranth	Sweet Potato	MT	PDR	SB	BD
A	72.97	18.92	8.11	0.70	32.92	124	358
B	76.74	16.28	6.98	6.43	38.33	89	395
C	67.35	26.53	6.12	0.51	33.75	101	108
D	62.79	30.23	6.98	1.14	40.00	57	56
E	70.13	18.18	11.69	0.70	33.75	56	166
F	74.16	15.73	10.11	7.50	40.00	18	228
G	65.35	25.74	8.91	0.54	37.92	59	106
H	60.67	29.21	10.11	0.49	37.92	42	95
I	64.52	25.81	9.68	0.59	30.83	57	122
J	71.79	20.51	7.69	0.46	37.08	135	305
K	77.42	12.90	9.68	5.05	40.83	70	268
L	61.54	30.77	7.69	0.49	40.00	39	49
M	70.59	23.53	5.88	0.70	35.65	112	128
N	66.67	22.22	11.11	0.68	33.75	63	196
O	68.57	22.86	8.57	0.50	33.33	89	141
O	68.57	22.86	8.57	0.50	33.33	91	190
O	68.57	22.86	8.57	0.50	33.33	95	183
O	68.57	22.86	8.57	0.65	33.33	95	178

MT: mixing time; PDR: peak dough resistance; SB: set back; BD: breakdown.

**Table 2:** Proximate composition of the wheat, amaranth and sweet potato flours.

Flour	Protein (%)	Lipids (%)	Moisture (%)	Ashes (%)	Carbo-Hydrates (%)
Wheat	11.6 ± 0.03 ^b^	1.50 ± 0.09 ^b^	8.98 ± 0.11 ^a^	0.76 ± 0.01 ^c^	77.18
Amaranth	17.9 ± 0.02 ^a^	7.35 ± 0.17 ^a^	3.62 ± 0.09 ^b^	2.85 ± 0.01 ^b^	68.24
Sweet potato	3.68 ± 0.08 ^c^	0.26 ± 0.01 ^c^	9.24 ± 0.12 ^a^	3.52 ± 0.02 ^a^	83.46

Results are expressed as mean ± SD of three determinations; different superscript letters within each column represent significant differences at *p* < 0.05.

**Table 3:** Significance and regression coefficients of the prediction models.

Variable	Intercept	X_1_	X_2_	X_3_	X_1_ * X_2_	X_1_ * X_3_	X_2_ * X_3_	R^2^	*p*-Value
MT	−7517.7	75.3	75.0	75.1	* −0.04	−0.02	-	0.73	0.004
PDR	24,588.0	* −245.5	* −245.5	* −245.8	* −0.08	0.05	−0.06	0.79	0.003
SB	167,407.0	−1671.5	−1674.2	−1683.6	* 0.63	0.90	1.47	0.76	0.006
BD	194,835.7	−1941.4	−1959.5	−1954.3	−0.07	-	* 4.98	0.86	0.000

MT: mixing time; PDR: peak dough resistance: SB: set back; BD: breakdown. X_1_: wheat, X_2_: amaranth, X_3_: sweet potato. * Significant terms of the response variable.

**Table 4:** Predicted optimized formulations of the blends.

	Ingredients (%)			Response Variables			
Blend	Wheat	Amaranth	Sweet Potato	MT	PDR	SB	BD
1	74	16	10	3.8	37.4	65.7	248
2	76	13	11	5	41.51	28.5	219
3	68.7	22.7	8.6	0.6	33.7	93	189
4	62.8	30.2	7.0	1.1	40	57	56

MT: mixing time; PDR: peak dough resistance; SB: set back; BD: breakdown.

**Table 5:** Specific volume and textural characteristics of the breads made with wheat flour and the optimal blends.

Bread	Specific Volume (cm^3^/g) **	Hardness (N) **	Cohesiveness *	Chewiness * (kg/mm)
Wheat	3.47 ± 0.3 ^ab^	9.04 ± 1.7 ^ab^	0.54 ^ab^	4.62 ^ab^
Blend 1	3.25 ± 0.2 ^ab^	10.44 ± 1.6 ^a^	0.53 ^ab^	5.20 ^ab^
Blend 2	3.21 ± 0.0 ^b^	10.72 ± 1.2 ^a^	0.51 ^b^	5.33 ^a^
Blend 3	3.59 ± 0.1 ^a^	6.66 ± 0.5 ^b^	0.56 ^a^	3.53 ^bc^
Blend 4	3.61 ± 0.1 ^a^	7.03 ± 1.7 ^b^	0.55 ^ab^	3.53 ^bc^

Different letters along the columns indicate significance (*p* < 0.05). ** Comparison by Tukey–Kramer test: mean ± SD. * Median comparison, non-parametric Kruskal–Wallis test (value z > 1.96): medians. In all the cases, the interquartile for cohesiveness was 0.512–0.557, and that for chewiness was 3.46–6.05.

**Table 6:** Proximate composition of the breads prepared with blend 3 and with 100% wheat.

Bread	Protein	Moisture	Ashes	Lipids	Carbo-Hydrates
Blend 3	10.15 ± 0.1 ^a^	38.1 ± 0.4 ^a^	2.44 ± 0.02 ^a^	3.0 ± 0.3 ^a^	46.28
Wheat	10.12 ± 0.2 ^a^	40.1 ± 0.1 ^a^	1.70 ± 0.05 ^b^	1.9 ± 0.0 ^b^	46.13

Results are expressed as mean ± SD of three determinations. Different superscript letters within each column represent significant differences at *p* < 0.05.

**Table 7:** Indispensable amino acid profile of blend 3 protein and the reference pattern.

Amino Acid (AA)	g AA/100 g Protein		
	Blend 3	Wheat *	Reference **
Ser + His	6.09 ± 0.10	6.89 ± 0.42	His 1.6
Thr	3.40 ± 0.06	2.70 ± 0.33	2.5
Met + Cys	3.75 ± 0.24	3.07 ± 0.37	SAA 2.3
Val	5.07 ± 0.01	4.03 ± 0.49	4.0
Phe + Tyr	7.60 ± 0.69	7.23 ± 0.88	AAA 4.1
Ile	4.06 ± 0.60	3.94 ± 0.48	3.0
Leu	5.42 ± 0.44	7.33 ± 0.89	6.1
Lys	2.98 ± 0.16	2.36 ± 0.29	4.8

Results are expressed as mean ± SD of three determinations. * Data from Hoehnel et al., 2020 [26]. ** FAO, 2013 [24]. SAA: sulphur amino acid (Met + Cys); AAA: aromatic amino acid (Phe, Trp, Tyr).

**Table 8:** Fiber, total phenolic compounds, β-carotenes, antioxidant capacity and glycemic index of the bread made with blend 3.

Compound or Property	Blend 3	Wheat
Dietary fiber (g/100 g)	4.98 ± 0.03	2.04 ± 0.03
Total phenolics (mg GAE/100 g)	83.13 ± 0.2	15.80 ± 0.3
β-carotenes (µg/100 g)	1123.2 ± 19.2	Traces
TEAC (mg TE/100 g)	242.4 ± 5.7	
DPPH (mg TE/100 g)	67.5 ± 2	6.22 ± 0.2
ORAC (mg GA/100 g)	15 ± 0	
Glycemic index	51.8	72.0

Results are expressed as mean ± SD of three determinations.

## Data Availability

Data is contained within the article.
